# The current state and development trends of frailty research in diabetic patients: a bibliometric analysis

**DOI:** 10.3389/fmed.2025.1529218

**Published:** 2025-03-11

**Authors:** Ziqi Xu, Rui Zhou, Xinran Zhou, Zhengyan Zhang, Qiong Li, Guodong Wang

**Affiliations:** ^1^School of Nursing, Xinxiang Medical University, Xinxiang, China; ^2^The First People's Hospital of Shangqiu City, Shangqiu, China; ^3^The Third Affiliated Hospital of Xinxiang Medical University, Xinxiang, China

**Keywords:** bibliometrics, diabetes, frailty, Cite Space, VOS viewer

## Abstract

**Background:**

Diabetes mellitus is a global public health issue, often leading to organ damage, complications, and disabilities. Frailty is an age-related syndrome characterized by reduced physiological reserve and increased vulnerability to stressors, significantly affecting the prognosis of older diabetic patients. The prevalence of frailty is notably higher in older adults with diabetes than in those without. Therefore, a bibliometric analysis of research on diabetes-related frailty can provide deeper insights into the current state of this field and inform future research directions.

**Methods:**

This study retrieved English-language publications on diabetes-related frailty from the Web of Science Core Collection (WOS) database, covering the period from 2005 to 2023. A total of 403 articles were included in the analysis. Statistical analysis and data visualization were conducted using Microsoft Excel, R Studio, VOS viewer, and Cite Space 6.3.R1. The analysis emphasized journals, authors, keywords, country collaborations, institutional collaborations, and references to elucidate trends and knowledge structures within the field of diabetes-related frailty research.

**Results:**

The number of publications on diabetes-related frailty has been steadily increasing each year, with research predominantly focused in developed countries, particularly the United States and Europe. The University of London has emerged as the institution with the highest volume of publications, while Alan J. Sinclair has been recognized as a significant contributor to this field. Key research hotspots include the complications associated with diabetes-related frailty, epidemiology, and quality of life. Additionally, a timeline analysis of references suggests that diabetic nephropathy is currently at the forefront of research in this area.

**Conclusion:**

This comprehensive bibliometric analysis of diabetes-related frailty research underscores the necessity for improved international collaboration to further investigate the mechanisms underlying diabetes-related frailty and to devise more effective prevention and treatment strategies. Future research should emphasize the relationship between diabetic nephropathy and frailty, as well as the development of personalized intervention programs tailored for frail diabetic patients.

## 1 Introduction

Diabetes represents a significant public health challenge that poses a serious threat to human health globally, resulting in considerable socioeconomic burdens worldwide (1). It is projected that by 2045, the number of individuals living with diabetes will reach 629 million worldwide (2). Diabetes is a metabolic disorder with multiple etiologies, characterized by chronic hyperglycemia stemming from defects in insulin secretion, insulin action, or both, which leads to disturbances in carbohydrate, fat, and protein metabolism (3). Prolonged hyperglycemia can result in chronic damage and functional impairment in various organs and tissues, including the eyes, kidneys, heart, blood vessels, and nerves, thereby severely impacting patients’ quality of life (4). Studies have shown that diabetes is associated with complications, disability, and frailty syndrome (5).

Frailty is a syndrome characterized by a decline in physiological function, primarily resulting from decreased physiological reserves. This decline leads to increased susceptibility to diseases, heightened vulnerability, and a diminished capacity to withstand stress, presenting as a nonspecific state (6). Currently, the concept of frailty is increasingly recognized as a multidimensional health condition, including physical frailty, cognitive frailty, psychological frailty, and social frailty (7).

Frailty is considered a novel complication in older patients with diabetes, affecting multiple systems and increasing the risk of adverse outcomes such as disability, hospitalization, and mortality (8). Furthermore, physical frailty has been demonstrated to correlate significantly with cognitive impairment, depression, and social vulnerability (9). Frailty and diabetes are two significant health issues commonly associated with aging in the older population. These conditions frequently co-occur and are becoming increasingly prevalent among older adults. As diabetes progresses and age advances, older patients with diabetes face a heightened risk of developing frailty, and the incidence of frailty is notably higher in this group. Research indicates that diabetic patients are more susceptible to frailty compared to their non-diabetic older counterparts (10). Therefore, early and timely assessment of frailty in older diabetic patients is of great clinical significance.

Research on the interplay between frailty and diabetes has received considerable attention. For example, Chhetri et al. (11) examined the current status and influencing factors of diabetes-related frailty through a longitudinal study. Shi et al. (5) investigated the effects of diabetes and frailty on mortality. Qin et al. (12) performed a meta-analysis to assess the efficacy of various exercise intervention modalities for patients with diabetes-related frailty. However, to date, there has been no notable bibliometric study assessing the knowledge mapping related to diabetes and frailty.

Therefore, this study employs bibliometric analysis to systematically examine the existing research on diabetes and frailty, with the aim of providing a comprehensive exploration of the relationships between diabetes and various aspects of frailty. This approach will help to more thoroughly grasp current research trends, the impact of the research, and scientific collaboration, to provide quantitative evidence to guide future research endeavors.

## 2 Methods and materials

### 2.1 Data sources and search strategy

In this study, all English language articles and reviews about diabetes and frailty, published between January 1, 2005, and December 31, 2023, were retrieved from the Web of Science (WOS) database on April 23, 2024. The search strategy utilized was: TS = (“diabetes mellitus*” OR diabetes* OR “diabetes disease” OR “diabetic mellitus” OR “diabetic disease”) AND (Frailty OR Frail*). A total of 1,453 publications were retrieved. After independent screening and discussion by two researchers, irrelevant publications were manually removed, resulting in a final total of 403 publications related to diabetes and frailty being included in the study analysis. All retrieved publications were exported from the online database in plain text format with full records and cited references.

### 2.2 Data analysis

Statistical analyses were conducted using Microsoft Excel 2021 and R Studio. Visualization analyses were performed using VOS viewer and Cite Space 6.3.R1. Microsoft Excel was primarily used to collect and analyze WOS data, create histograms, and build regression models to predict publication growth trends. Data extraction and statistics, including main information, most relevant authors, most influential journals, and most globally cited documents, were conducted by the “bibliometrix” package in R Studio (13).

VOS viewer software demonstrated remarkable potential in the field of bibliometric analysis. Its generated visual maps, characterized by their simplicity and comprehensibility, effectively transform complex information into intuitive knowledge frameworks, providing researchers with unprecedented insights (14). VOS viewer was utilized to create visual analyses of author collaboration maps and keyword clustering maps.

Cite Space facilitates identifying the developmental progress and trends within research fields by analyzing titles, abstracts, and references (15). Cite Space 6.3.R1 was used to visually analyze international collaboration maps, institutional collaboration network maps, dual-map overlays of journals, keyword burst maps, and reference timeline maps. In these visual knowledge maps, different nodes represent elements such as countries, institutions, authors, or cited references; links between nodes represent relationships such as collaboration, co-occurrence, or co-citation; the colors of the nodes and lines represent different years. Centrality measures the importance of nodes in the paths connecting any pair of nodes in the network. The parameters for Cite Space were set as follows: (1) the time span was divided from January 2005 to December 2023, with each slice representing 1 year; (2) term sources = title/abstract/author keywords/keywords plus; (3) node types (one at a time) and selection criteria (top 50 objects); (4) selecting the top 10% most cited items from each slice.

## 3 Results

### 3.1 Publications

**Figure 1:**
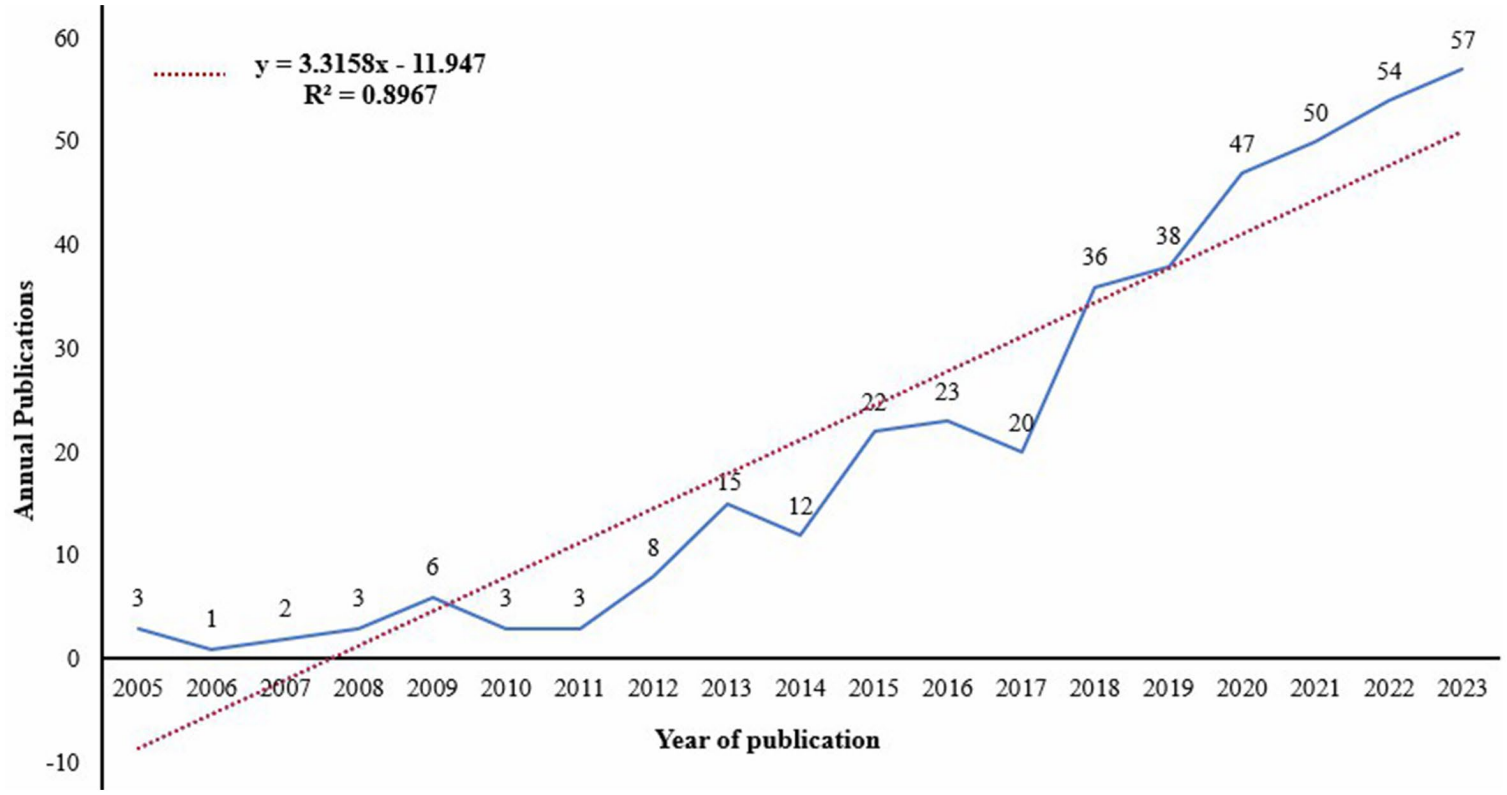
Rend of publication volumes for clinical practices on diabetes and frailty from 2005 to 2023.

**Figure 2:**
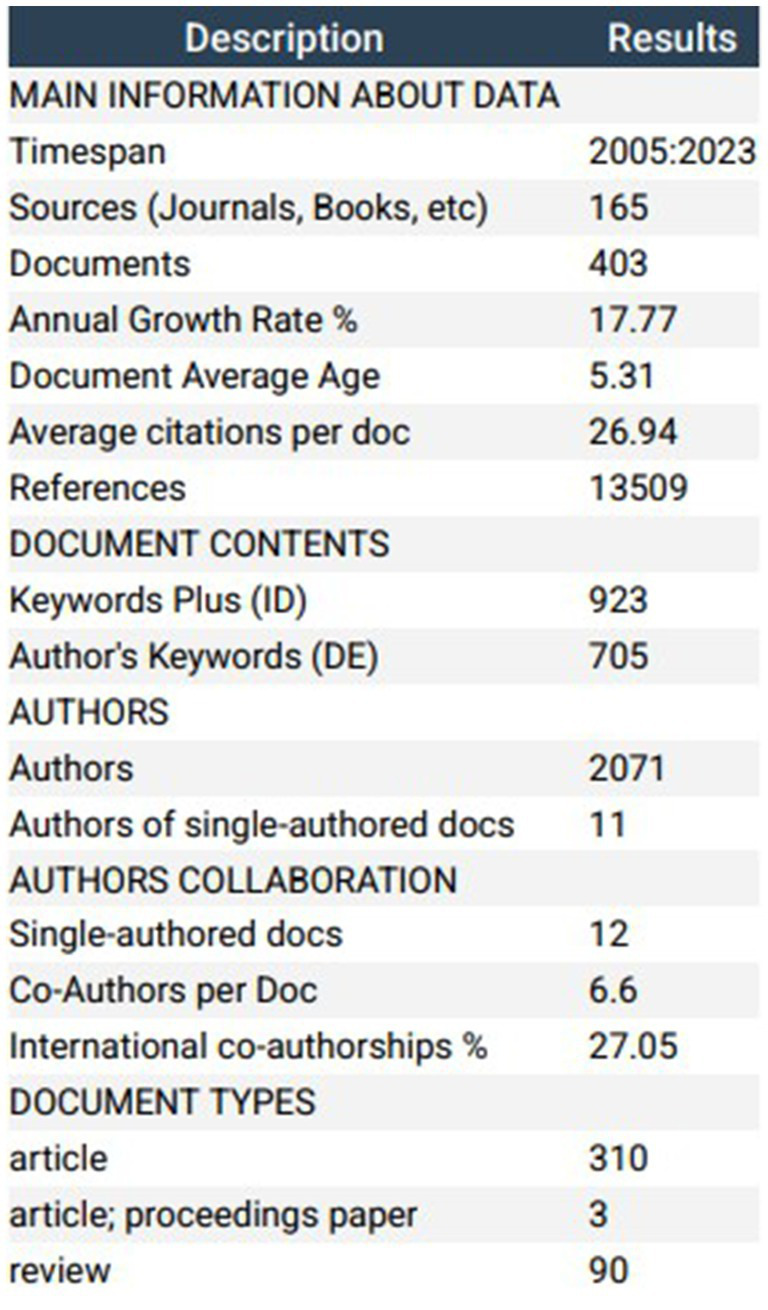
Key information summary of all articles from 2005 to 2023.

This study utilized the citation report and citation deduplication functions within the Web of Science (WOS) database to analyze 403 articles related to diabetes and frailty published between 2005 and 2023. An Excel-generated publication trend chart, illustrated in Figure 1, reveals a consistent year-on-year increase in publications from 2005 to 2017, followed by a marked surge from 2017 to 2023, culminating in the highest publication count of 57 in 2023. To further elucidate the publication volume trend, a linear trend line equation was established: y = 3.3158x−11.947, where y signifies the annual publication volume and x represents the year. The coefficient of determination (*R*^2^) for this model is 0.8967. Figure 2, generated by bibliometrix, provides a comprehensive overview of the analyzed articles, which collectively cite 13,509 references, with an average publication year of 5.31 years. Each article received an average of 26.94 citations, and the annual growth rate of publications was 17.77%.

### 3.2 Countries and regions

**Figure 3:**
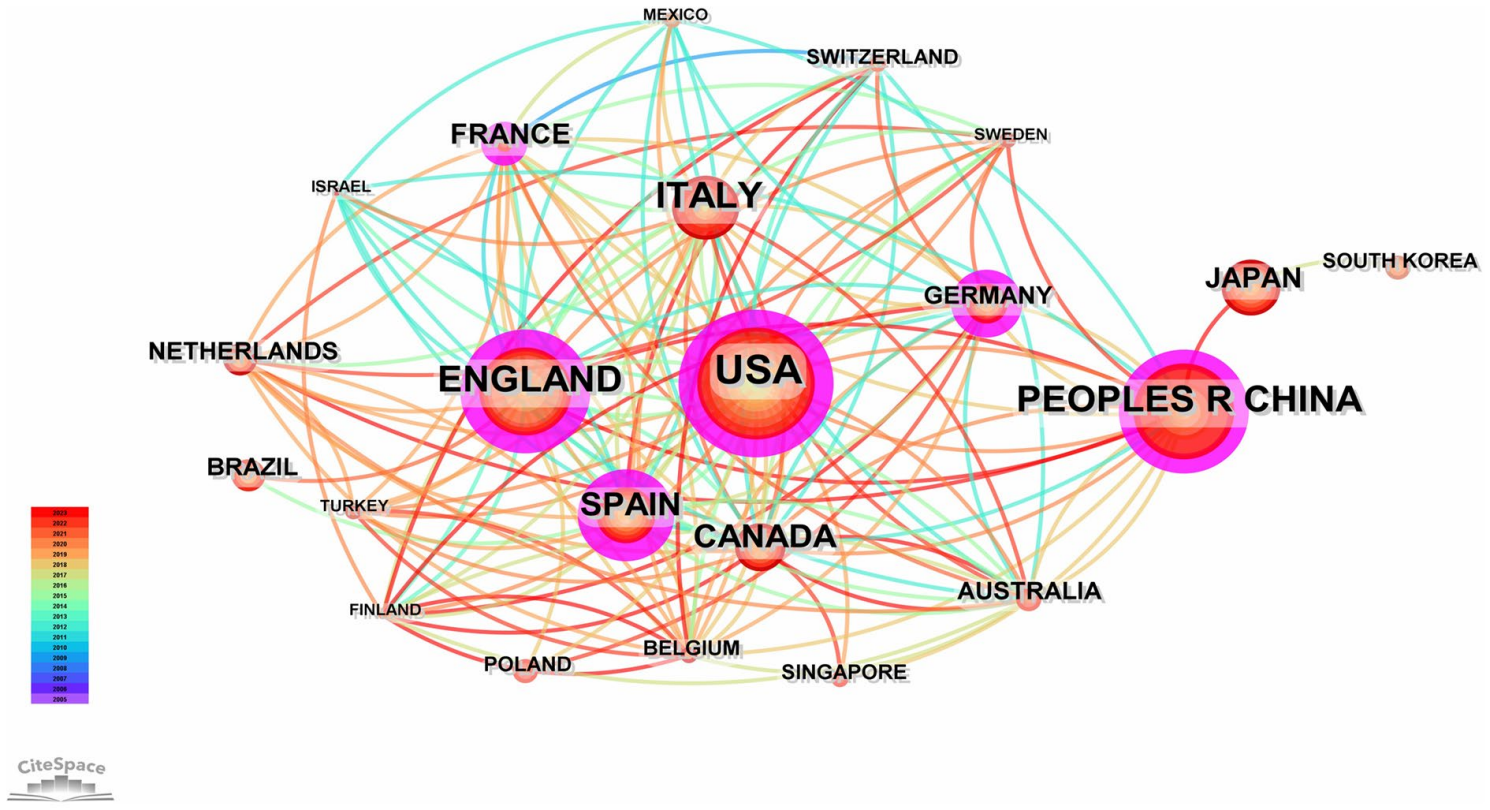
Network map illustrating international collaboration among countries publishing articles related to diabetes and frailty between 2005 and 2023.

Through the use of Cite Space, it was found that between 2005 and 2023, research on diabetes and frailty was conducted across 50 countries. As illustrated in Figure 3, the size of each label reflects the volume of publications, indicating that the top three countries in terms of publication output are the United States, the United Kingdom, and China. The purple circles outside the labels represent centrality, where a larger centrality signifies greater influence, suggesting that countries with strong centrality in this topic area include the United Kingdom, the United States, Spain, Germany, China, and France. Table 1 presents statistics on the top 10 countries in terms of publication volume.

**Table 1:** Top 10 countries publishing articles on diabetes and frailty from 2005 to 2023.

Rank	Country/Region	Counts, *n* (%)	Centrality
1	USA	99 (24.57)	0.26
2	England	75 (18.61)	0.33
3	China	61 (15.13)	0.15
4	Italy	55 (13.65)	0.03
5	Spain	45 (11.17)	0.21
6	Canada	34 (8.44)	0.04
7	Japan	30 (7.44)	0.07
8	France	28 (6.95)	0.14
9	Australia	18 (4.47)	0.02
10	Germany	16 (3.97)	0.18

### 3.3 Institutions

**Figure 4:**
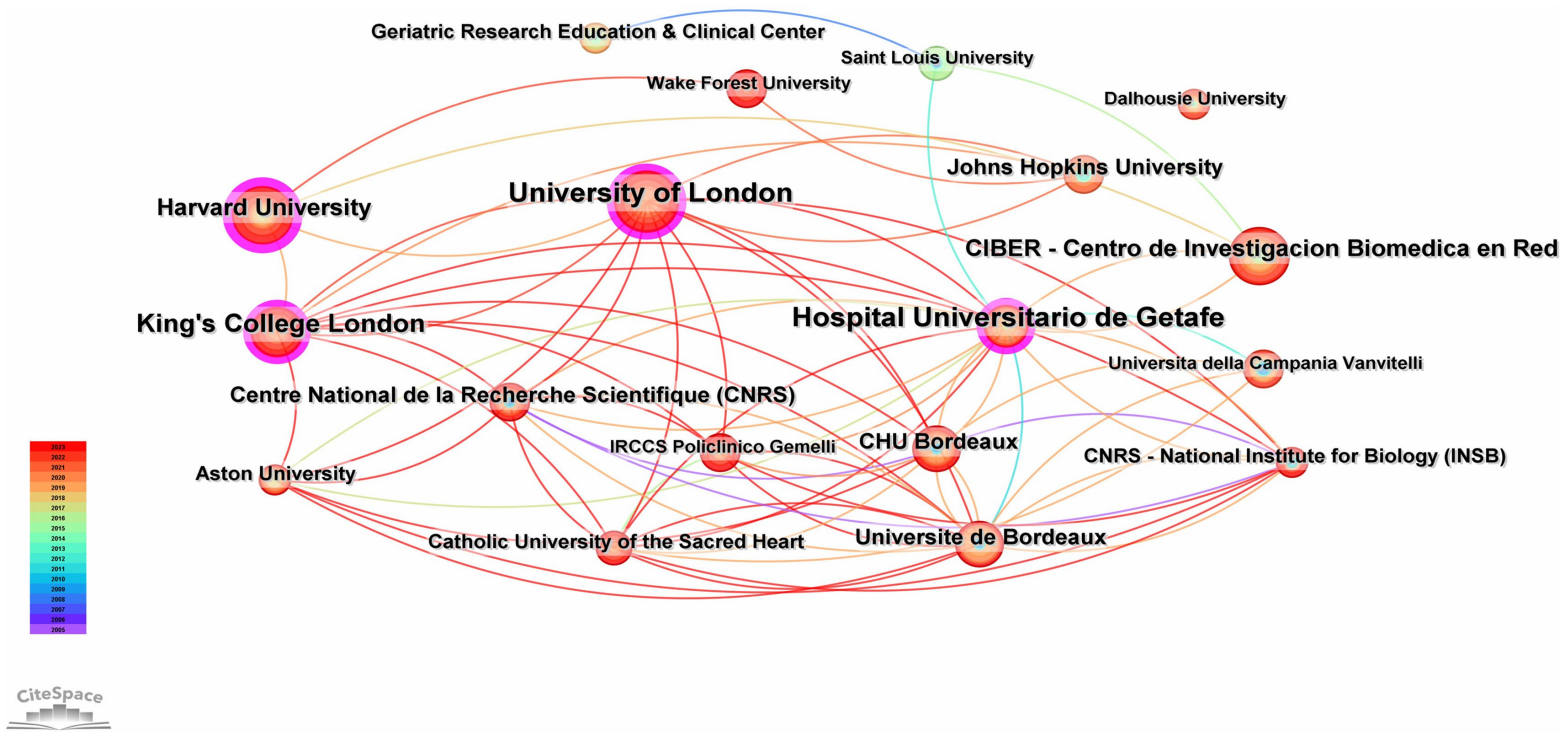
Collaboration network analysis of institutions involved in clinical practices related to diabetes and frailty from 2005 to 2023.

A total of 288 institutions have engaged in research on diabetes and frailty. Figure 4, generated by Cite Space, illustrates that the University of London leads in publication volume in this field, followed by Hospital Universitario de Getafe and King’s College London. Notably, institutions with higher centrality, indicating significant influence, include Harvard University, Hospital Universitario de Getafe, Assistance Publique-Hôpitaux de Paris, University of London, and King’s College London; the lines in the figure denote collaborative relationships between institutions. Table 2 ranks institutions that have published more than 10 articles, with a total of 7 institutions having published more than 7 articles, among which there are 2 each from the United Kingdom, France, and Spain, and one from the United States.

**Table 2:** Institutions with more than 10 publications on diabetes and frailty from 2005 to 2023.

Rank	Institutions	Count	Centrality	Country/Region
1	University of London	22	0.16	England
2	Hospital Universitario de Getafe	21	0.17	Spain
3	King’s College London	19	0.13	England
4	Harvard University	15	0.22	USA
5	CIBER—Centro de Investigación Biomédica en Red	15	0.09	Spain
6	Universite de Bordeaux	12	0.02	France
7	CHU Bordeaux	12	0.01	France

### 3.4 Analysis of authors

**Figure 5:**
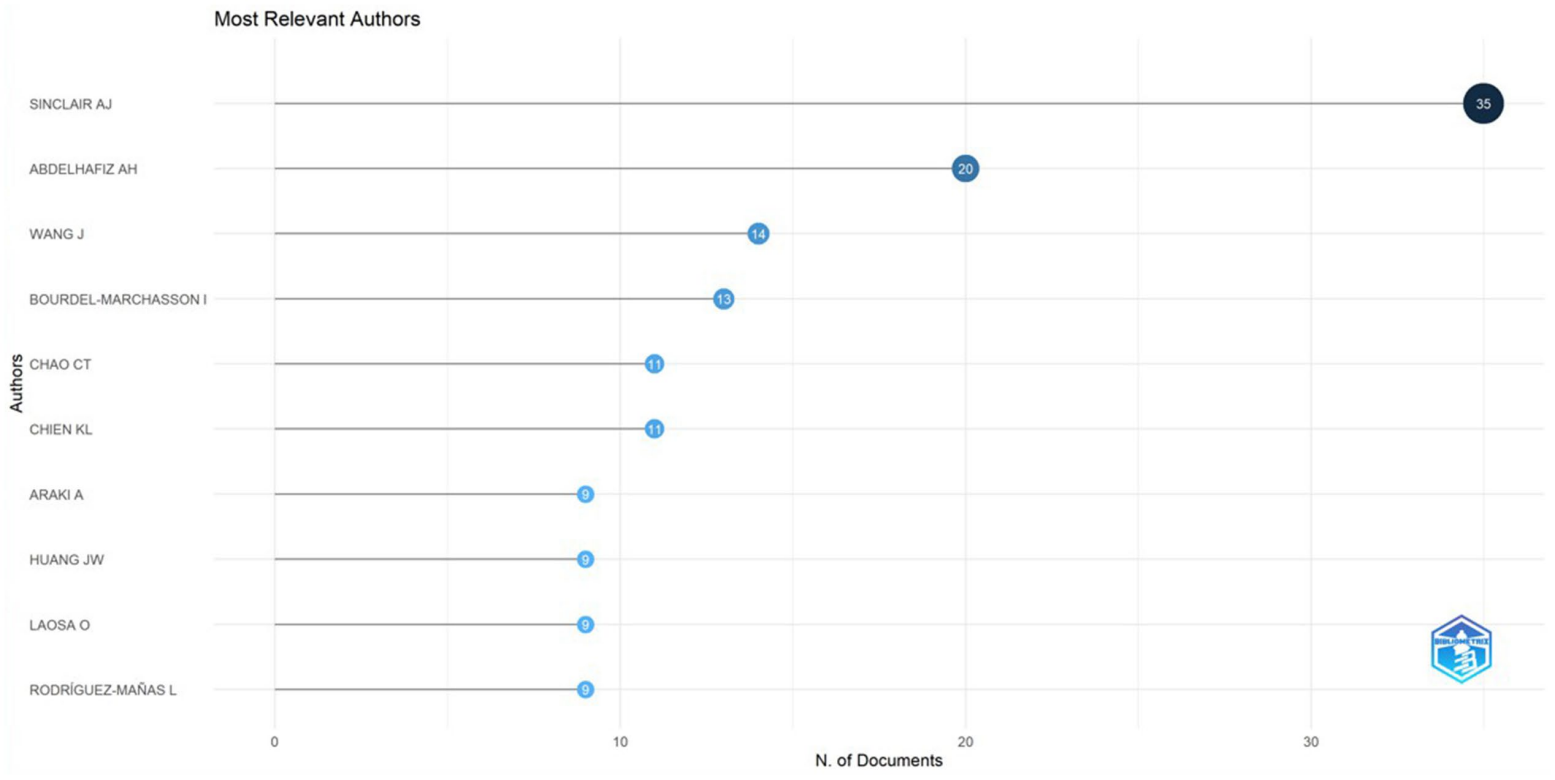
The top 10 most relevant authors in the field of diabetes and frailty.

**Figure 6:**
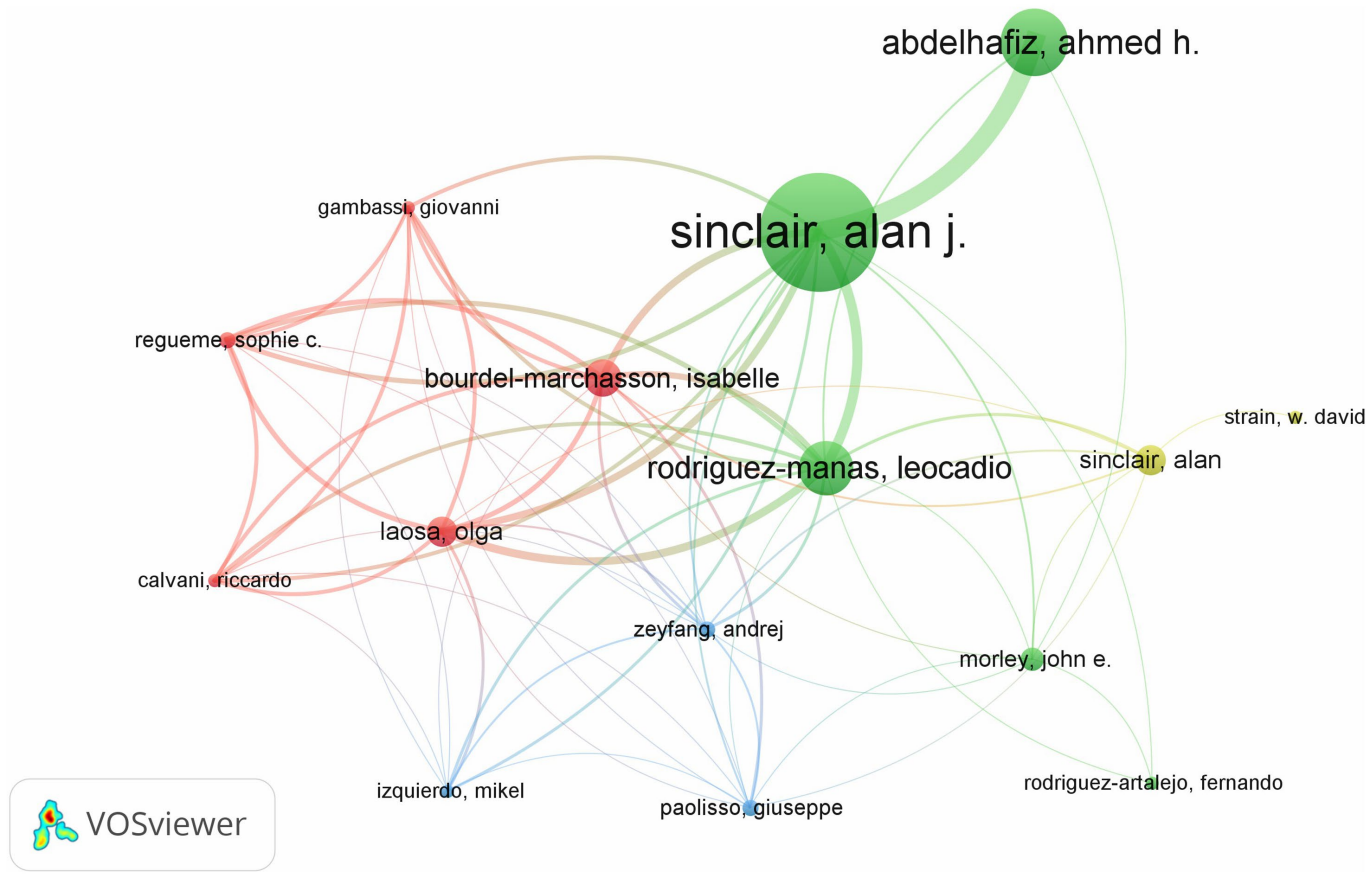
Network map depicting the collaborations among authors in the field of diabetes and frailty research.

**Figure 7:**
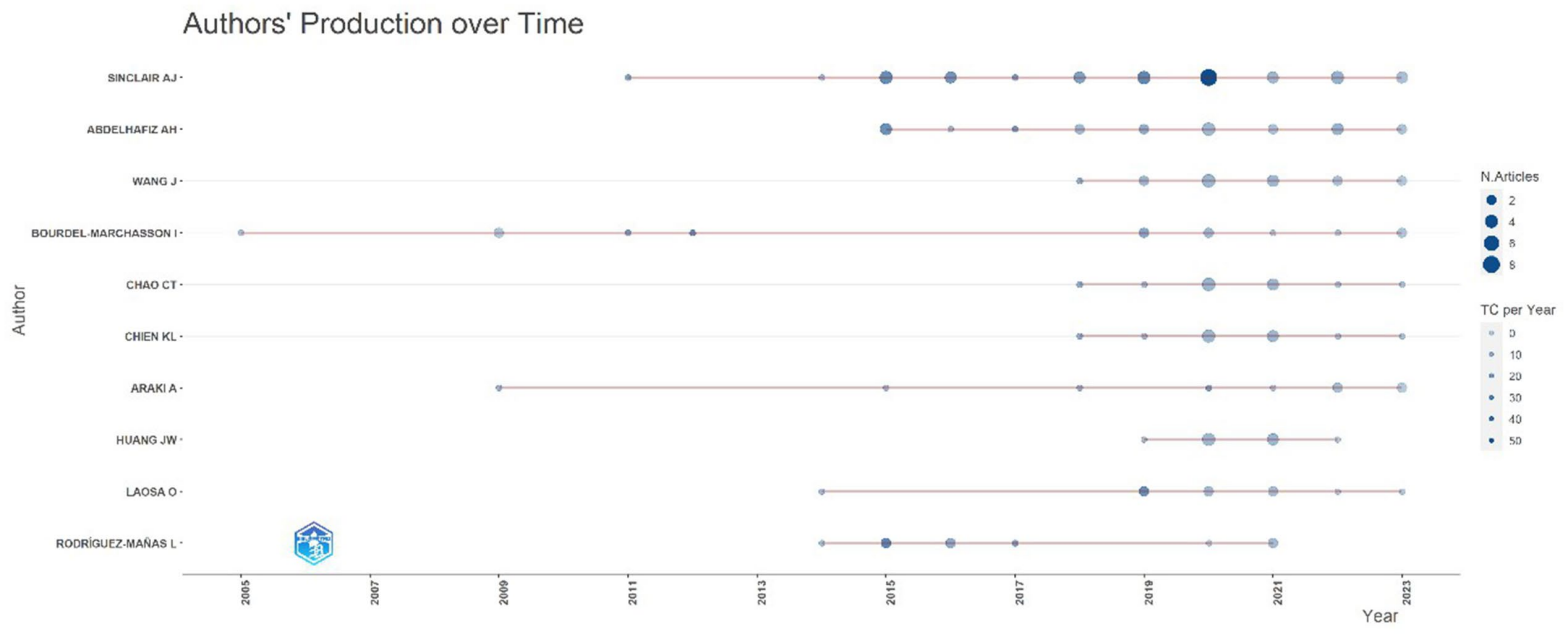
Dynamic chart showing the publication volume over time for the top 10 authors with the highest number of publications.

**Figure 8:**
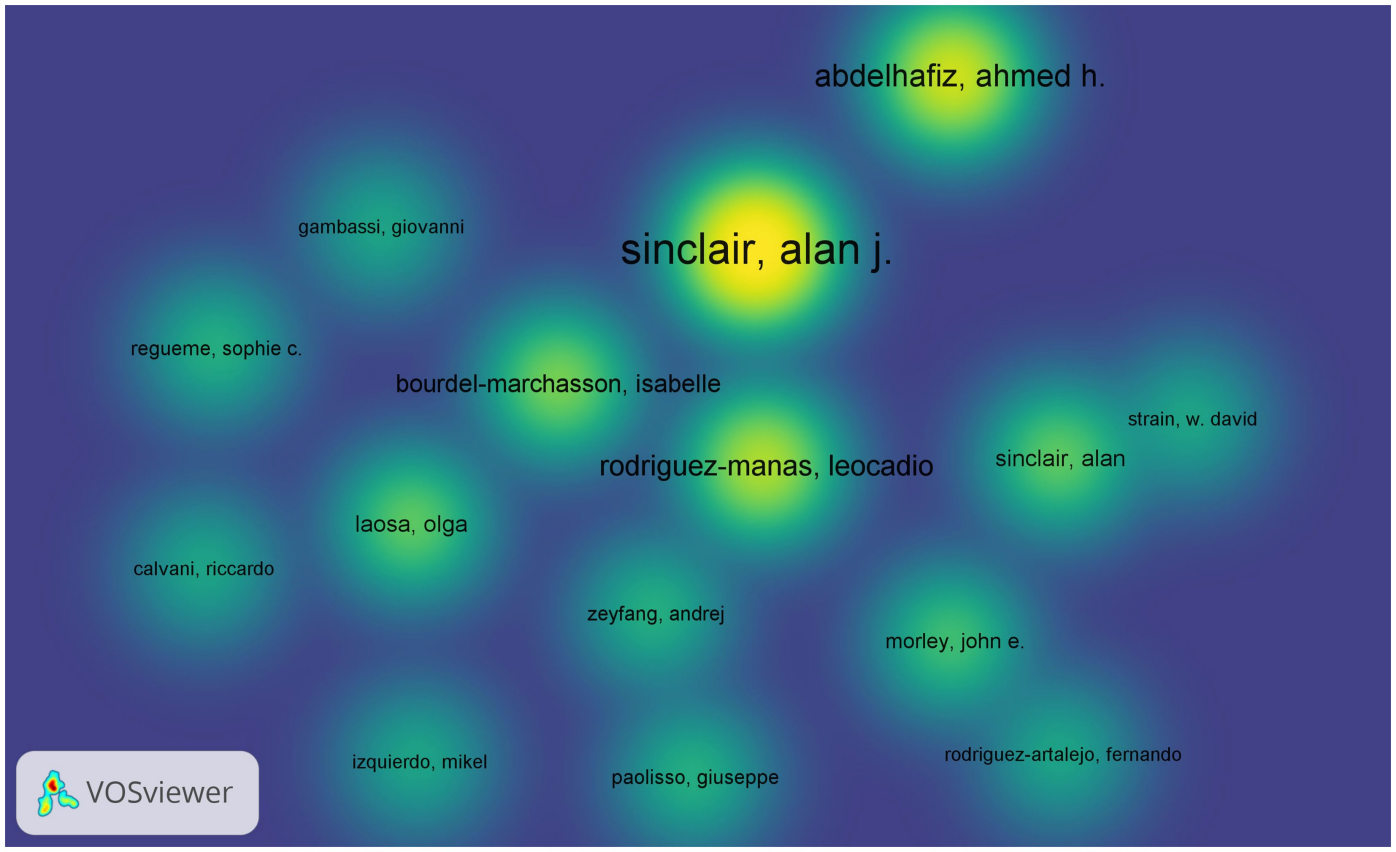
Heat map representing the activity of authors in the field of diabetes and frailty research.

Figure 5, generated by bibliometrix, presents the top 10 most prolific authors who have published articles in the field of diabetes and frailty between 2005 and 2023. Alan J. Sinclair is the author with the highest volume of publications in this field, having published a total of 35 articles. According to Price’s Law for calculating the minimum number of publications for highly productive authors (*m* = 0.749), the minimum publication threshold for highly productive authors is calculated to be 4.4 papers. Consequently, the minimum number of publications for authors displayed by VOS viewer is set at 4, and after manual deduplication, the result is shown in Figure 6. Four distinct color clusters are formed, where nodes of the same color belong to the same cluster, and the shorter the distance between nodes, the stronger the association. Figure 7, generated by bibliometrix, is a dynamic publication chart over time for the top 10 authors, where the size of the circle represents the number of documents, and the depth of the color indicates the total number of citations. Figure 8, generated by VOS viewer, is a heat map of author activity in this field, where the deeper the color of the node label, the stronger the research impact, and Alan J. Sinclair is the most influential author in this domain.

### 3.5 Journals

**Figure 9:**
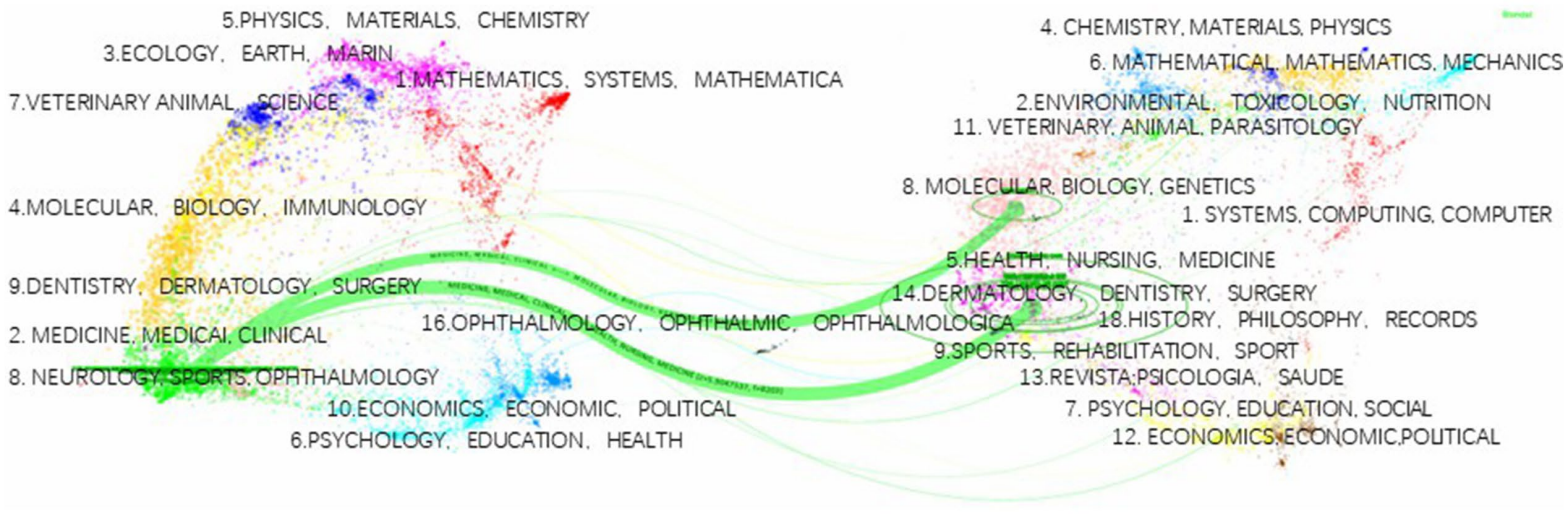
Dual-map overlay spectrum of journals in the field of clinical practice research on diabetes and frailty.

Bibliometrix analysis was employed to identify influential journals in the field of diabetes and frailty by examining citing and cited journals. As shown in Table 3, among the citing journals, Journals of Gerontology Series A-Biological Sciences and Medical Sciences ranks highest, followed by BMC Geriatrics, with impact factors of 5.1 and 4.1 respectively; the journal with the highest impact factor among citing journals is Journal of the American Medical Directors Association. Among the cited journals, Diabetes Care leads the list, followed by J Gerontol A-Biol and J Am Geriatr Soc, with impact factors of 16.2, 5.1, and 6.3 respectively; the journal with the highest impact factor among cited journals is the Lancet. A dual-map overlay created through Cite Space is presented in Figure 9. The left side, labeled in blue, represents the research fields of citing journals, while the right side, labeled in purple, represents the research fields of cited journals. The lines connecting the labels on either side indicate the citation relationship between citing and cited journals in their respective research fields. Two prominent green intersecting curves in the diagram suggest that journals in the fields of Molecular Biology, Genetics, and Health Nursing Medicine are more likely to be cited by journals in the field of Medicine, Medical, Clinical.

**Table 3:** Top 10 journals cited in research on diabetes and frailty.

Rank	Citing journals	Count	2023IF	Rank	Cited journals	Count	2023IF
1	Journals of Gerontology Series A-Biological Sciences and Medical Sciences	17	5.1	1	Diabetes Care	1,626	16.2
2	BMC Geriatrics	15	4.1	2	J Gerontol A-Biol	937	5.1
3	Journal of Nutrition Health & Aging	14	5.8	3	J Am Geriatr Soc	887	6.3
4	Journal of the American Medical Directors Association	14	7.1	4	J Am Med Dir Assoc	676	7.6
5	Journal of Diabetes and its Complications	13	3	5	New Engl J Med	558	158.5
6	Geriatrics & Gerontology International	12	3.3	6	Lancet	475	168.9
7	Aging Clinical and Experimental Research	10	4	7	Age Aging	324	6.7
8	Diabetic Medicine	10	3.5	8	J Nutr Health Aging	318	5.8
9	Journal of the American Geriatrics Society	10	6.3	9	JAMA—J Am Med Assoc	318	120.7
10	Diabetes Research and Clinical Practice	9	5.1	10	Diabetes Res Clin Pract	291	5.1

### 3.6 Keywords

**Figure 10:**
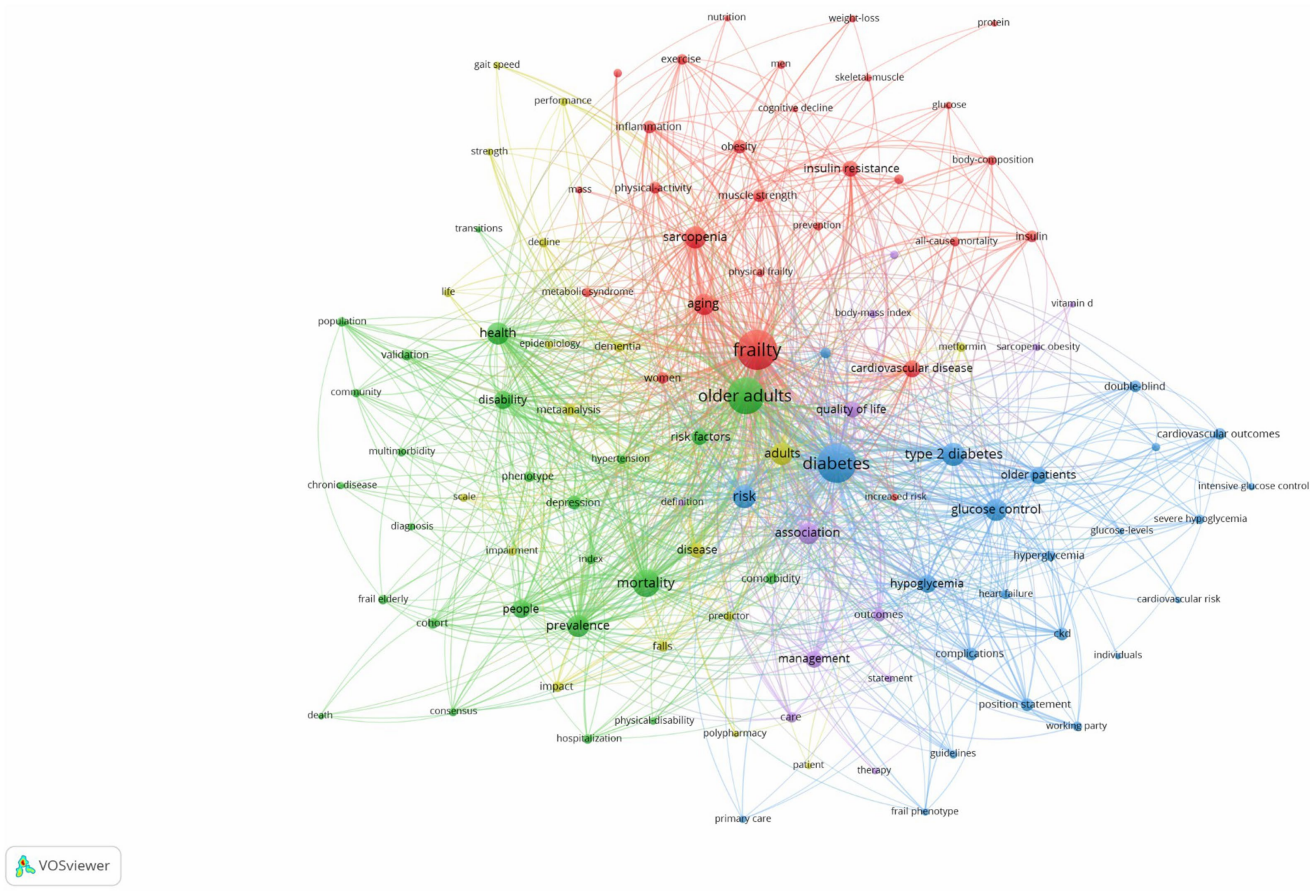
Cluster map of keywords in the field of diabetes and frailty research.

A frequency analysis of the top 100 keywords appearing in the research field of diabetes and frailty was conducted using VOS viewer. After deduplication and merging of similar keywords, a threshold of 7 occurrences was set, leading to the selection of 107 high-frequency keywords for further analysis. As illustrated in Figure 10, keywords with the same color denote that they belong to the same cluster, resulting in the formation of five distinct clusters within this domain. By analyzing the relationships among these keywords in Figure 10, three prominent research themes can be identified: complications, epidemiology, and quality of life. The red cluster focuses on complications associated with frailty, such as sarcopenia, obesity, cardiovascular disease, and insulin resistance, emphasizing their role in the mechanisms of frailty in diabetic patients. The blue cluster primarily addresses glycemic control issues in type 2 diabetic patients, including hypoglycemia and hyperglycemia, and is concentrated on related complications such as cardiovascular disease and nephropathy. The green cluster pertains to epidemiological studies, featuring key terms like mortality, prediction, and risk factors. Both the yellow and purple clusters concentrate on quality of life but from different perspectives. The yellow cluster predominantly looks at dementia, cognition, and falls, which are related to personal health status, whereas the purple cluster emphasizes management, outcomes, and care, focusing on the overall management aspects of quality of life.

**Figure 11:**
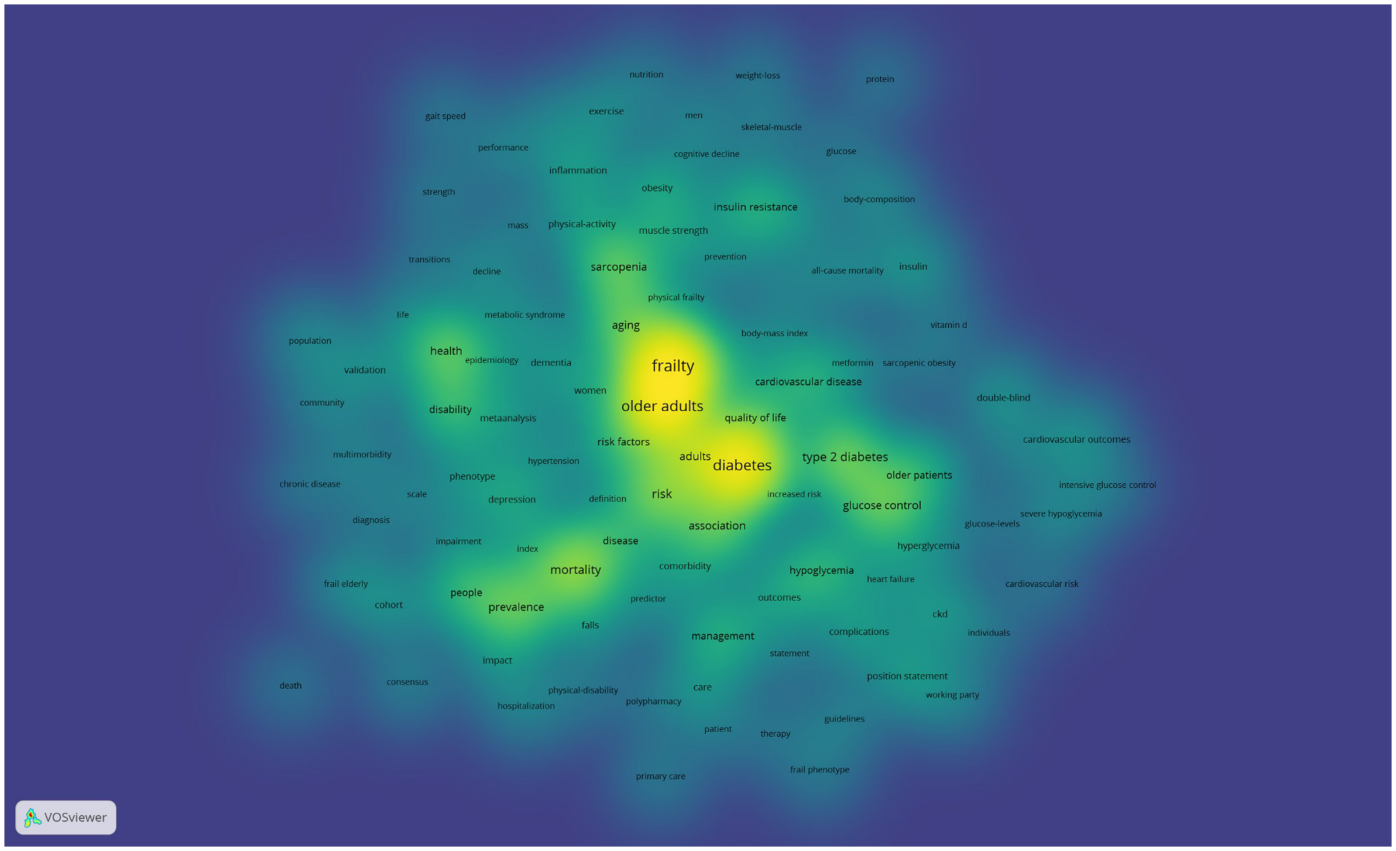
Heat map of keywords in the field of diabetes and frailty research.

**Figure 12:**
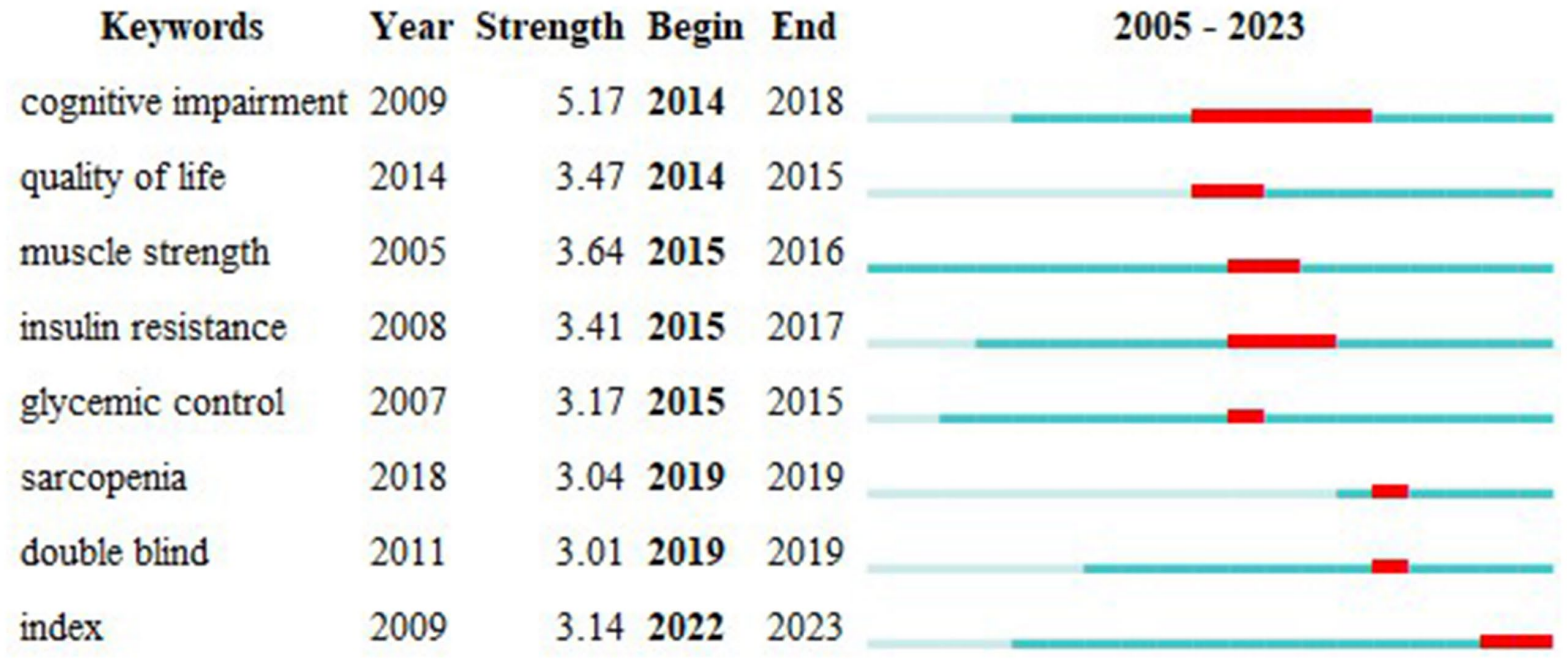
Burst keyword chart in the field of diabetes and frailty research.

Figure 11 presents the keyword heat map, where brighter colors indicate higher frequencies of keyword occurrence, and closer proximity to the center suggests higher citation and co-citation frequencies. Table 4 lists the top 10 most frequent keywords, with “Frailty” and “Diabetes” being directly relevant to the topic, while “Older adults” and “Mortality” have high frequencies, indicating past hot topics. To pinpoint the current frontiers in research, a burst keyword map was generated by Cite Space, as shown in Figure 12. Six burst keywords are listed on a timeline, with “cognitive impairment” being the strongest burst term, and “index” representing the primary focus of current research, potentially exerting significant influence on future studies.

**Table 4:** Top 10 high-frequency keywords in the field of diabetes and frailty research.

Rank	Keywords	Counts
1	Frailty	234
2	Diabetes	220
3	Older adults	193
4	Mortality	105
5	Type 2 diabetes	81
6	Risk	80
7	Prevalence	74
8	Adults	73
9	Sarcopenia	71
10	Glucose	70
10	Health	70

### 3.7 Co-cited references

**Figure 13:**
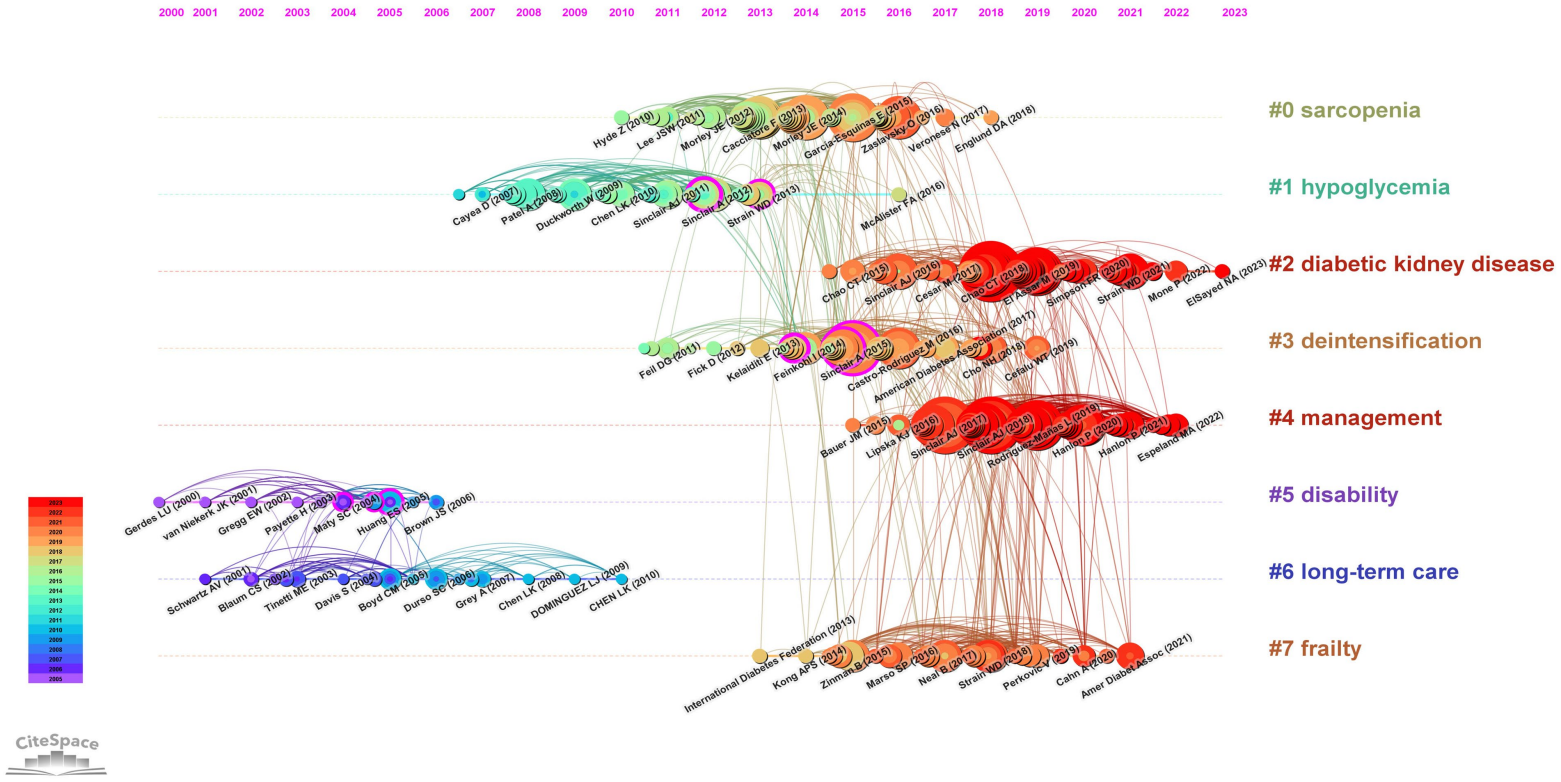
Timeline view of referenced literature in the field of diabetes and frailty research.

By conducting a timeline view analysis of co-citation relationships among references using Cite Space, we can gain insights into the thematic evolution and developmental trajectory of research in diabetes and frailty. Clustering analysis of references based on keywords yields the results depicted in Figure 13. On the left side of Figure 13, the co-citation relationships among references are displayed over time, with the size of the nodes representing the frequency of citation and the color of the nodes indicating the time of citation. On the right side, the clustering outcome is presented, where clusters with labels positioned closer to the front denote greater cluster strength. “#Sarcopenia” emerges as the most significant cluster, and “#Diabetic Kidney Disease” has continued to be a focus up until 2023. Statistical analysis was conducted using bibliometrix, Table 5 enumerates the top 10 most cited reference documents.

**Table 5:** Top 10 most cited references in the field of diabetes and frailty research.

Rank	Title	Times cited
1	Frailty in older adults: evidence for a phenotype (66)	199
2	Frailty in elderly people (67)	50
3	Frailty, sarcopenia and diabetes (68)	45
4	A global clinical measure of fitness and frailty in elderly people (69)	44
5	Diabetes and risk of frailty and its potential mechanisms: a prospective cohort study of older adults (70)	42
6	Accelerated loss of skeletal muscle strength in older adults with type 2 diabetes (71)	38
7	Frailty and sarcopenia – newly emerging and high impact complications of diabetes (72)	38
8	Clinical frailty and long-term mortality in elderly subjects with diabetes (73)	37
9	Prevalence of frailty in community-dwelling older persons: a systematic review (74)	37
10	Intensive blood glucose control and vascular outcomes in patients with type 2 diabetes (75)	37

## 4 Discussion

### 4.1 Main information

Over the past 19 years, the number of academic articles in the field of diabetes and frailty has generally shown an upward trend. Before 2017, the growth in publication volume was relatively gradual, but post-2017, there was a significant increase. This growth could be attributed to the increasing attention of researchers to the study of diabetes and frailty. As shown in Figure 7, in 2017, three of the top 10 authors in terms of total publications emerged. By 2018, this number increased to six, with three authors publishing their first academic articles in this field in 2018.

The analysis of Figure 7 also indicates that international collaboration is predominantly concentrated among high-income Western countries, with the United States having the highest publication volume and the United Kingdom exhibiting the highest centrality, making it the most influential country in this field. These two countries have jointly led research in this area. Among middle- and low-income countries, only China has conducted relevant research and has closely collaborated with high-income countries. This is likely because China has the highest number of people with diabetes globally, necessitating more research efforts in this area. In Asia, only China, Japan, Singapore, and South Korea have engaged in limited collaboration (16).

In summary, research in the field of diabetes and frailty is uneven, with middle- and low-income countries lacking in contributions to this field. Asian countries should engage in more collaborative efforts and strengthen cooperation with high-income Western countries to improve research levels and international influence. Figure 9 depicts the primary research areas of citing and cited journals in this field, indicating the flow of research outcomes and showing that the primary research areas have a narrow coverage. Future research needs to span multiple disciplines, and there is considerable potential for further exploration.

This study aims to comprehensively analyze the literature in the field of diabetes and frailty, explore research hotspots, and identify future development trends by clustering keywords and references to recognize the key research areas and directions. Analyzing highly cited literature revealed that three out of the top 10 references focus on frailty assessment, indicating inconsistencies in assessing frailty among diabetic patients. Additionally, three articles study the correlation between frailty and sarcopenia in diabetic patients, highlighting a research hotspot in this field, which is confirmed by clustering analysis results.

### 4.2 Variations in frailty assessment among diabetic patients

Current research on diabetes and frailty primarily focuses on type 2 diabetes, with limited studies addressing frailty in older individuals with type 1 diabetes. Type 1 and type 2 diabetes are distinct diseases, and their relationship with frailty may differ. At present, the management of frailty in older individuals with type 1 diabetes is inferred from studies on type 2 diabetes patients (17). Although the American Diabetes Association (ADA) guidelines provide some guidance on the management of older individuals with type 1 diabetes, there is a need for further research to explore the unique aspects of frailty in this population and develop tailored management strategies (18). Therefore, future studies should focus on investigating the specific characteristics of frailty in older individuals with type 1 diabetes and the corresponding management approaches.

Frailty assessment should be completed for all older diabetic patients as part of diabetes management (19). Early identification of frailty, assessment of frailty levels, and timely intervention can significantly delay the progression of diabetes and its related complications (20). Some experts recommend routine comprehensive geriatric assessments (CGA), including frailty assessments, for the older diabetic population (21). To date, there is no unified, universally accepted concept or “gold standard” for the measurement of frailty internationally (22). The most commonly used assessment tools are the frailty phenotype (FP) proposed by Fried and the frailty index (FI) proposed by Rockwood (23).

FI is a direct application of the cumulative deficit frailty index, which quantifies frailty through an index composed of several equal-weighted deficits from different domains, including physical, functional, psychological, and social aspects (24). Additionally, the Montreal Cognitive Assessment (MoCA) and the Subjective Cognitive Decline Questionnaire (SCD-Q) are commonly used for the assessment of cognitive frailty (25, 26).

### 4.3 Impact of complications on frailty in diabetic patients

Older diabetic patients are prone to chronic long-term complications, which cause neurological, vascular, and metabolic abnormalities, leading to muscle fatigue (27). This results in muscle weakness, slow gait, and eventually frailty. Research indicates that sarcopenia is the core pathological basis of frailty, with skeletal muscle loss playing a mediating role in its development (28). Chronic hyperglycemia in diabetic patients can inhibit the growth of skeletal muscle cells, leading to muscle atrophy. Additionally, insulin resistance can hinder glucose uptake by skeletal muscle cells, resulting in muscle contraction disorders, causing sarcopenia, and accelerating the onset of frailty (29). Therefore, early identification of sarcopenia in diabetic patients is crucial for reducing the incidence of frailty syndrome.

Frailty is closely associated with the readmission rates, mortality, and postoperative complications in older cardiovascular disease (CVD) patients, serving as a significant predictor of adverse clinical events and outcomes. The occurrence and development of CVD and frailty mutually reinforce each other, sharing similar pathogenesis (30, 31). Frailty predicts poor prognosis in cardiovascular disease patients, often leading to a reduced ability to respond to external stimuli and increasing the likelihood of various adverse clinical events (32). Hence, healthcare providers should emphasize the screening and early assessment of frailty in older CVD patients. Prompt and effective targeted interventions for frail patients can help reduce or delay the occurrence of severe adverse outcomes.

### 4.4 Epidemiological studies on frailty in diabetic patients

Current research on diabetes and frailty primarily focuses on the older population. Due to differences in geographic location, research environments, and frailty assessment tools, the reported prevalence rates vary widely, ranging from 7.5 to 56.7% (29, 33). A meta-analysis revealed that the global prevalence of frailty among older diabetic patients in the community is as high as 20.1%. In Europe, North America, and South America, the prevalence rates are 21.7, 24.9, and 22.1%, respectively, with Asia having the lowest at 14.3% (34). This indicates that the incidence of frailty among older diabetic patients is relatively high. In addition, multiple studies on older diabetic patients have found that the prevalence of frailty is higher in women than in men (33, 35). Therefore, early screening and intervention for frailty should be integrated into diabetes care for the older to prevent and mitigate its adverse effects.

Frailty is closely associated with various adverse health outcomes in diabetic patients, including hospitalization, disability, and death. Frailty can increase the risk of hospitalization and mortality in diabetic patients and significantly elevate the risk of disability (36, 37). However, the underlying mechanisms linking frailty to adverse health outcomes in diabetic patients remain unclear. Frailty may be closely related to reduced physical activity, leading to poor prognosis (38). Additionally, increased inflammation and coagulation abnormalities during frailty may exacerbate the microvascular effects of diabetes, thereby increasing the incidence of complications, hospitalization rates, and mortality (39).

Biological studies show that both depression and frailty in the older are associated with vascular lesions, white matter changes, elevated levels of tumor necrosis factor, and interleukin-6 (40). Moreover, frailty itself implies a decline in physiological function, limited daily activities, and increased caregiving needs. When patients perceive changes due to frailty, they may experience an identity crisis and increased physical and psychological stress, thereby raising the risk of depression (41). Frailty can accelerate the progression of diabetes. Research indicates that elevated serum levels of soluble receptor for advanced glycation end-products (sRAGE) can predict mortality in frail older individuals (42).

### 4.5 Quality of life in frail diabetic patients

Compared to non-diabetic individuals, diabetic patients exhibit decreased cognitive abilities and face an increased risk of dementia and mild cognitive impairment (43). Although diabetic patients can effectively control blood glucose levels and prevent complications through strict self-management, cognitive decline may reduce their ability to self-manage. The concept of cognitive frailty refers to the coexistence of physical frailty and mild cognitive impairment in the absence of dementia or other pre-existing neurological conditions (44, 45). For patients with diabetes, the occurrence of cognitive frailty may further exacerbate the challenges of self-management, as they may struggle to effectively cope with disease management and treatment while simultaneously facing declines in both physical and cognitive functions.

A survey of type 2 diabetic patients over 40 years old found a significant negative correlation between frailty and self-management behaviors, particularly in diet, physical activity, and medication adherence. Frail diabetic patients are more likely to develop diabetes-related complications; studies also indicate that individuals with higher blood glucose levels are more prone to cognitive impairment, leading to difficulties with multiple and continuous medications (46, 47). Metformin, a common medication for diabetes, is supported by clinical studies for its potential benefits in extending lifespan and as an anti-aging drug (48). Pan Liu and colleagues found in a study of 422 type 2 diabetic patients over 40 years old that metformin is negatively correlated with frailty, suggesting that frailty may reduce the long-term protective effects of metformin. Early identification and intervention for frailty in diabetic patients may enhance the efficacy of metformin (49). The decline in physical activity due to frailty may also serve as a risk indicator for cognitive decline. A study found that among frail diabetic patients, those with a 4-meter walking time over 4 s exhibited significant declines in verbal fluency, indicating that gait speed could serve as a risk indicator for cognitive decline (50).

As age increases, frailty elevates the risk of adverse clinical outcomes in the older. Diabetic patients, who often have complex care needs, require special attention to frailty issues (51). Frailty is associated with an increased risk of a range of negative health outcomes in diabetic patients, including microvascular complications, impaired activities of daily living, mortality, cardiovascular events, hypoglycemia, and hospitalization. It can also predict falls and readmission in older diabetic outpatients (52, 53). Frailty in diabetic patients is linked not only to physical health decline but also to declines in psychological health and quality of life. A study indicated that frail diabetic patients had significantly lower quality of life assessments and higher severity of depressive symptoms compared to non-frail diabetic patients (54). Another study found that frail diabetic patients had higher levels of frailty, more severe depressive symptoms, and lower quality of life scores compared to non-diabetic frail individuals (55). Complications and adverse reactions to treatment in diabetic patients complicate treatment plans, making comprehensive management essential for improving their quality of life.

For older diabetic patients, frailty assessment should be a routine part of their evaluations to determine treatment plans and blood glucose control goals. The dynamic nature of frailty necessitates regular assessments, as eliminating hyperglycemia and hypoglycemia can improve frailty (56). Intensive blood glucose control targets may harm frail diabetic patients, and glucose management should focus on reducing hypoglycemia symptoms and simplifying medication regimens (57). Continuous glucose monitoring (CGM) can provide real-time, continuous blood glucose level monitoring, offering more comprehensive information on glucose fluctuations. This can help doctors formulate more personalized and precise treatment plans and enable patients to better understand their glucose patterns, enhancing their glucose management awareness and self-regulation capabilities (58, 59). Therefore, CGM may have significant application value in managing frail diabetic patients. In conclusion, addressing frailty in diabetic patients requires incorporating frailty assessments into routine management, developing individualized intervention plans, and optimizing comprehensive management strategies to delay frailty progression and improve patient quality of life.

### 4.6 Frontiers in frailty research among diabetic patients

As illustrated in Figure 13’s reference timeline, Cluster #2 (diabetic kidney disease, DKD) represents the current research frontier, indicating future research directions in this field. The chronic course of diabetes often leads to microvascular complications such as diabetic nephropathy (DN), which has become increasingly relevant in the study of diabetes-related frailty (60). DKD is a progressive condition, primarily caused by diabetes, and is a form of chronic kidney disease. Clinically, it is often characterized by proteinuria and a progressive increase in serum creatinine levels (61). Current research indicates a bidirectional relationship between DKD and frailty in diabetic patients. DKD is a significant risk factor for frailty in diabetic patients, while frailty impacts the clinical outcomes and overall health status of patients with DKD (62).

A follow-up study of 322,109 newly diagnosed diabetic patients over a median period of 2.89 years revealed that those with chronic kidney disease (CKD) before diabetes diagnosis had the highest probability of developing frailty, being 2.597 times more likely than those without DKD. Diabetic patients who developed DKD post-diagnosis had a 2.137 times higher likelihood of frailty compared to those without DKD. Over time, the risk of frailty increases in those with pre-diabetes CKD, highlighting the potential significance of early frailty intervention in the early stages of diabetes (63). Hormonal changes in diabetic patients, such as deficiencies in vitamin D, insulin-like growth factor-1, and testosterone, contribute to bone fragility and subsequent osteoporosis. A propensity score-matched study of 24,054 DKD patients and 12,027 DKD patients with osteoporosis revealed an increased risk of frailty in DKD patients with osteoporosis, suggesting a link between frailty and adverse outcomes in these patients (64). DKD can cause adverse symptoms such as headaches and limb pain, leading patients to use muscle relaxants for symptom relief. A propensity score-matched study of 11,637 long-term muscle relaxant users and 11,637 non-users among DKD patients found that prolonged use of muscle relaxants increased the risk of frailty, indicating that reducing muscle relaxant use may lower frailty risk in DKD patients (65). Most current research is correlational, predominantly cohort studies, lacking experimental studies to establish a causal relationship between DKD and frailty. Future research should focus on high-quality studies to explore this causality. The relationship between DKD and frailty is complex and bidirectional, forming a vicious cycle. Future research should emphasize interdisciplinary collaboration to explore the mechanisms between DKD and frailty and develop effective interventions tailored to the frailty characteristics of different DKD patients.

## 5 Limitations and future research directions

The limitations of this study include the fact that the literature search was conducted exclusively in the WOS database, which may have resulted in the omission of relevant studies that are indexed in other databases. However, due to the varying number of publications across different databases, it is not feasible to merge data from multiple sources. The analysis in this study did not involve the selection of a specific age range, resulting in variability in the age groups of the included study populations. Furthermore, there is a limited understanding of frailty across these diverse age groups. A limitation of the current study is the relatively limited focus on frailty in individuals with type 1 diabetes, as the majority of research has concentrated on type 2 diabetes. Furthermore, there is a lack of studies addressing the social and psychological dimensions of frailty in the context of diabetes. These aspects remain underexplored and represent valuable areas for future research, offering a more comprehensive understanding of frailty and its impact on diabetic patients. Addressing these gaps would provide a deeper insight into the multifaceted nature of frailty and enhance management strategies for diabetic patients, particularly those with type 1 diabetes.

## 6 Conclusion

This study found that the number of scholars focusing on the field of diabetes and frailty has increased year by year, with the primary research centers located in the United States and Europe. The University of London stands out as the institution with the most publications, and Sinclair AJ is identified as the most significant contributor to this research field. This study summarized the research hotspots in diabetes and frailty, focusing on frailty screening, the impact of complications on diabetic frailty patients, epidemiological studies of diabetic frailty patients, and their quality of life. The research frontier primarily concerns issues related to diabetic kidney disease (DKD). This bibliometric analysis of publications from the past 19 years in the field of diabetic frailty is of significant importance for future research.

## Data Availability

The raw data supporting the conclusions of this article will be made available by the authors, without undue reservation.
